# Non-medical use of olanzapine by people on methadone treatment

**DOI:** 10.1192/pb.bp.115.052886

**Published:** 2016-12

**Authors:** Philip David James, Ali Shaik Fida, Pavel Konovalov, Bobby P. Smyth

**Affiliations:** 1 Health Service Executive, Louth/Meath, Ireland; 2 Chilliwack, British Columbia, Canada; 3 Dublin North City and County CAMHS, Dublin, Ireland; 4 Department of Public Health and Primary Care, Trinity College Dublin, Ireland; 5 Health Service Executive, Dublin, Ireland

## Abstract

**Aims and method** We examined non-medical use (NMU) of olanzapine among adults on methadone treatment. Information was collected on patient demographics and NMU of olanzapine. The Alcohol, Smoking and Substance Involvement Screening Test (ASSIST) was administered to assess risk among current users of olanzapine.

**Results** Ninety-two clients participated and 30% reported lifetime history of NMU of olanzapine. Nine people reported doses of 30 mg or higher on a typical day of use, with three typically using 100 mg. The most common reasons for use were to relieve anxiety and to aid sleep, but a quarter used it to ‘get stoned’. Eleven participants (12%) reported NMU of olanzapine in the preceding month. Eight completed the ASSIST with four scoring in the high-risk zone.

**Clinical implications** Self-medication is the dominant motivator for NMU of olanzapine, but hedonic motivations also occur. A small minority show features of dependency. All doctors should be aware of the potential NMU of olanzapine, especially among patients with history of addiction.

Globally, there is a long history of non-medical use of prescription drugs (NMUPD), evident across many sectors of society.^1–3^ Opioid analgesics and benzodiazepines are the drug classes most frequently encountered in studies of NMUPD.^1,2,4^ The past 20 years have witnessed a move towards non-medical use (NMU) of an increasing spectrum of medications, including the newer hypnotics,^5^ methylphenidate^6^ and antidepressants.^7^ People who engage in NMUPD against a background of polysubstance use tend to use more regularly and in larger quantities than those who engage in NMUPD alone.^4^ Various terms are encountered in the literature that describe situations where medications are being used without a valid prescription from a medical practitioner, such as non-medical use, non-prescription use, abuse and misuse. For the purpose of this article we will use the term non-medical use (NMU).

Antipsychotics are among the medications most commonly prescribed by psychiatrists but evidence, typically single case studies, has emerged regarding their NMU. One of the earliest reports involves psychiatric patients who appear to have become dependent on their antipsychotic medications, including haloperidol and trifluoperazine.^8^ In a study of adolescents attending addiction treatment in Ireland it emerged that NMU of antipsychotics exceeded that of stimulant medications for attention-deficit hyperactivity disorder (ADHD).^3^ Quetiapine appears to be the most commonly encountered antipsychotic appearing in reports on NMU, used orally, intranasally and intravenously.^9–12^

Literature also suggests that NMU of olanzapine may occur. A case report outlines NMU of olanzapine for anxiolytic effects and euphoric effects and to manage cocaine-related adverse effects.^13^ Further case reports describe apparent withdrawals and psychological dependence on prescribed olanzapine.^14,15^ In light of increasing reports in the literature and anecdotal reports of patients in our clinical practice reporting NMU of olanzapine, we decided to conduct a study to assess its prevalence among patients on methadone maintenance treatment (MMT).

## Method

### Setting

We conducted a survey of adult patients attending the National Drug Treatment Centre (NDTC) in Dublin. Patients attending from one clinical team in the NDTC, which provides treatment to about 130 individuals, were included. They were invited to participate in the survey on a voluntary basis.

### Instruments

We developed a structured questionnaire to explore past and recent NMU of olanzapine and what motivated it. This was informed by previous research on NMUPD. The questionnaire was administered via interview and took about 15 min to complete. It provided information on basic demographics, psychotropic medication use, illicit substance use, and health and psychosocial issues related to the study aims. Participants were also given the opportunity to provide additional qualitative information to questions and the data were analysed and categorised by two of the authors (P.J. and B.S.).

The Alcohol, Smoking and Substance Involvement Screening Test (ASSIST) is a World Health Organization (WHO) structured clinical interview.^16^ For each substance category, questions are asked about the 3-month period prior to interview. This then generates a risk score for that substance which ranges from 0 to 39. A score over 27 indicates high-risk use and that a specific addiction treatment response is required. A score of 4 to 26 indicates moderate risk use and a brief intervention is advised. We applied the ASSIST questions to olanzapine in cases where a person reported any NMU of olanzapine in the month before the interview.

## Results

We interviewed 92 people, which constituted about 70% of the eligible group. Information was not recorded on patients who were unavailable, uninterested or unwilling to participate. The respondents were predominantly male (*n*=60, 65%), were on methadone a mean of 5 years and had a mean age of 31 years. Twenty-eight (30%) reported a lifetime history of NMU of olanzapine. They did not differ significantly from those without a history of use in terms of gender, age or duration of methadone treatment.

Of participants reporting a lifetime history of NMU of olanzapine, two reported currently being prescribed this medication and only taking it as prescribed. Information on patterns of use was missing for one person. The recent use characteristics of the remaining 25 people are outlined in Table 1. Nine people reported doses of 30 mg or higher on a typical day of use, three of whom reported using 100 mg. The three heaviest users of olanzapine consumed 900 mg, 1200 mg and 1800 mg, respectively, in the month preceding the interview.

**Table 1:** Characteristics of 25 patients reporting non-prescription olanzapine use

	*n*	Median	%	IQR
Currently prescribed olanzapine	3		12	

Describes tolerance^a^	9		41	

Describes compulsion to use^b^	9		37	

Experience of adverse effects or problems with olanzapine use^b^				
None	2		8	
Excessive cost	10		42	
Feel tired and sick	19		79	
Feel sleepy most of the time	18		75	
Loss of interest in sex	7		29	
Do not want to go to work	11		46	
Loss of appetite	0		0	
Cannot stay focused	11		46	
Feel nauseous or uncomfortable	1		4	

Positive impact of olanzapine^b^				
None	0		0	
Give me the high I like	1		4	
Feel less worry or fear	18		75	
Sleeping better	23		96	
More interest in sex	4		17	
Better appetite	15		62	
Less pain	4		17	
Relieves side-effects of methadone	4		17	
Any NMU of olanzapine in past month?^b^	11		46	
Days used in past month among users		15		4–30
Number of uses per day		2		1–3
Total dose olanzapine on typical day, mg		20		15–60
Total dose consumed in past month, mg		400		200–600

NMU, non-medical use.

a. Data missing in three cases.

b. Data missing in one case.

Of the 11 people reporting current NMU of olanzapine, 8 completed the modified ASSIST: 4 had scores indicating high-risk use (range 29–35) and the other 4 had scores in the moderate risk zone (range 13–19).

All 28 people who reported that their first use of olanzapine was prescribed indicated why it was prescribed. Three of these were vague answers. Of the remaining 25 participants, 19 (76%) reported that they had been prescribed it to treat psychosis or a psychotic symptom (e.g. paranoia, delusions). Depression was mentioned by 4 (16%), anxiety by 3 (12%) and anger/agitation and bipolar affective disorder (BPAD) by 2 (8%) participants.

In addition to the list of possible reasons for NMU of olanzapine presented in the questionnaire (Table 1), clients were asked to name any additional reasons why they used it; 7 (25%) specifically stated ‘to get stoned’. All those who reported NMU of olanzapine were also asked what happens if they miss a dose. Again, all 28 answered this question, with 8 (29%) indicating that missing a dose had no effect or they never took it regularly enough to notice any effect. The most common effect of missing a dose was feeling anxious or nervous, which was reported by 14 participants (50%). The next most common effects were feeling angry or agitated (*n*=5, 18%), paranoid or psychotic (*n*=3, 11%), low mood (*n*=2, 7%) and feeling frustrated (*n*=2, 7%).

## Discussion

This is the first study to formally examine the extent of NMU of olanzapine. The finding that 30% of the clients surveyed reported NMU supports the evidence from previous case studies that it is a medication with misuse potential. Of those engaged in current use who completed the ASSIST, all had scores that indicated some risk, with half in the high-risk/probable dependence category.

The most common reasons for NMU of olanzapine were to relax or relieve tension, to aid sleep or simply to escape from worries. This is consistent with the reports from case studies.^13,15^ Two of the published case studies describe a euphoric feeling or a ‘high’ from olanzapine.^13,15^ A quarter of those who engaged in NMU of olanzapine used it to ‘get stoned’. Therefore our findings support a self-medication style of NMU of olanzapine as the dominant motivator for use, with hedonic motivations being present in a minority. The self-medication hypothesis has been proposed as a major precipitator and perpetuator of substance use in heroin-dependent populations.^17^ By comparison, the published case studies on NMU of quetiapine seem to involve clients using it for its euphoric effects,^8,11,12^ but this is not exclusively the case.^10^

This study has a number of limitations. It was completed within one clinic in one city and findings may not generalise to other settings. Although the ASSIST is a validated instrument, the questionnaire used in this study has not been assessed for reliability or validity. Furthermore, the study relied on self-reported use of olanzapine and was not independently verified by any objective biological testing method.

In the previous case studies published on olanzapine all the patients appear to have been prescribed it for varying psychiatric conditions and all were in contact with psychiatric services.^13–15^ While our findings and the previous case reports suggest caution when prescribing olanzapine to people with a history of dependence, there is also growing evidence that olanzapine is effective in the treatment of individuals with schizophrenia and a co-occurring substance use disorder.^18^ Similarly, there is evidence that quetiapine may also be effective specifically in helping those with substance use disorders.^19,20^ There are also studies indicating that other antipsychotics including aripiprazole,^21^ risperidone^22^ and clozapine^23–25^ have positive effects on a variety of substance use-related outcomes such as cravings and relapse. Petrakis *et al*,^26^ however, reported that atypical antipsychotics had no superior benefit in substance use outcomes when compared with traditional antipsychotics.

A small number of patients were using olanzapine in very large quantities. Olanzapine, and other antipsychotic medications, carry a large number of potential side-effects. There is no information on the particular risks of long-term very high-dose olanzapine use. The advice is that olanzapine should be used with caution in particular among individuals with hepatic impairment.^27^ Chronic hepatitis C is highly prevalent in this patient group and this is frequently compounded by high-risk alcohol use.^28,29^ Consequently, compromised hepatic functioning may pose particular hazards for people on methadone treatment and NMU of olanzapine. Those working within substance use services need to be mindful of the potential for NMU of antipsychotic medications and be cautious when considering introducing these medications. Since this study was completed, it has emerged that olanzapine was detected in 6.3% of all poisoning deaths in Ireland in 2012, twice as many as 3,4-methylenedioxymethamphetamine (MDMA).^30^ While clinicians worry about the increased appetite, weight gain and possible metabolic syndrome which can occur with therapeutic doses of olanzapine, it is noteworthy that over three-fifths of those who engaged in NMU of olanzapine cited improved appetite as a desired and positive effect of taking this drug, particularly as drug users frequently complain of weight loss and poor appetite.^31^

In light of our findings, and the literature in general, a great deal more research is needed to ascertain the extent of NMU of antipsychotics. While there is a need to be aware of risks, doctors treating individuals with medications that have misuse potential should not become paralysed by a fear of NMU as this could cause ill patients to get substandard care.^32^

Our findings indicate that NMU of olanzapine occurs in a significant minority of patients on methadone treatment. Further research is warranted to ascertain the level of NMU of other antipsychotics and among other patient groups. It appears that NMU is mainly motivated by a desire to get relief from a variety of unpleasant symptoms, although some individuals use it for hedonic effects. Staff working with substance users ought to be vigilant to the potential NMU of antipsychotics as these substances are not detected in standard drug screening and can contribute to adverse medical outcomes. All doctors should be aware of the potential NMU of olanzapine, especially among patients with a history of addiction.
